# How does the ‘care of the self’ impact on fitness to practice in dental education?

**DOI:** 10.15694/mep.2019.000139.1

**Published:** 2019-06-20

**Authors:** Melanie Hayes

**Affiliations:** 1 The University of Sydney

**Keywords:** Foucault, dental education, reflection, fitness to practice.

## Abstract

This article was migrated. The article was marked as recommended.

Foucault defines care of the self as activities which “permit individuals to effect by their own means or with the help of others a certain number of operations on their own bodies and souls, thoughts, conduct, and way of being, so as to transform themselves in order to attain a certain state of happiness, purity, wisdom perfection, or immortality” ( Foucault, 1988). This paper will explore how the care of the self impacts on fitness to practice in dental education. Fitness to practice refers to a student’s competence, including clinical skills and professionalism as well as the ability to reflect on their own health and capabilities. This paper will examine how the dominant discourse of reflective practice influences a student’s care of the self using techniques of writing and verbalisation, and how the development of competent graduates is tied to Foucault’s work on disciplinary power, panopticism and governmentality. The role of pastoral power and confession, and the relationship of educators and students will also be explored.

## The regime of truth: reflective practice

The current dominant discourse in dental education centres on reflective practice, as evidence of professionalism, competence and ultimately fitness to practice. Reflective practice has become a “cornerstone of pedagogy in professional preparation” ( Nelson, 2012) in health education, including dentistry. Thus, it has become what Foucault referred to during his exploration of governmentality as a regime of truth, a discourse which is accepted as true and is reinforced by institutions ( Foucault, 1991). This concept explores “how diverse types of knowledge, expertise and practices are developed..(that play out as) rules of the game for responsible conduct” ( Ng, 2016). Foucault also described episteme as the implicit foundations of our knowledge and what we consider to be the truth ( Holmes *et al.,* 2008). Despite Foucault describing the difficulty with getting “caught up in conventional ways of thinking” ( Foucault, 1996), he also acknowledged that care of the self is not only “knowledge of the self..but also knowledge of a number of rules or acceptable conduct” ( Foucault, 1997). In his archaeological exploration of technologies of the self, Foucault identified that engaging in reflective practice was perceived as a positive activity ( Foucault, 1988), an ethical manifestation of the care of the self.

## Reflective writing and care of the self

Care of the self allows one the freedom to question themselves and their surroundings, which in turn gives one the capacity to live ethically and care for others ( Batters, 2011); it allowed one to develop mastery of their chosen skill as well as encourage social responsibility ( Ng, 2016; Darbyshire and Fleming 2008). During the Hellenistic period, writing was one of the main activities for taking care of oneself ( Foucault, 1988). Letters written by Marcus Aurelius demonstrate how the details of everyday life were written in an examination of the conscience ( Foucault, 1988). In dental education, these include activities such as reflective writing, blogs, and self-assessment of practical experiences. Such activities are designed to empower students to explore and evaluate the learning or experience that has taken place, so that they may intentionally transform their practice, thus becoming self-organised learners ( Nelson, 2012).

Through care of the self and reflective practice, Foucault reasoned that individuals were able to make ethical and moral judgements and actions even if these contradicted what they understood to be normal ( Mackey, 2007). This may result is parrhesiastic activity, where one is frank in their convictions, and tells the truth as an apparent duty despite a power imbalance (Foucault, 1983). While there is certainly an opportunity for dental students to use reflective writing as a truth-telling exercise, it is questionable whether they are truly parrhesiastic in this activity. Students are encouraged to be honest and critical in order to develop their competency, but may prefer to reflect on safe topics in fear of offending their examiner and negatively impacting on their grades. Students tend to focus on problems they have encountered, and offer potential solutions or ways to improve the outcome in future similar situations, particularly concerning patient communication or clinical techniques.

Care of the self becomes critical when developing positive relationships with patients or clients ( Batters, 2011). In order to create trusting relationships with others, one must have the ability to reflect on themselves and their surroundings. In his archaeological exploration of the Romans and the Stoics, Foucault concluded that the care of the self is necessary when caring for others ( White, 2014); he remarked that it was important for one’s relationships with family, friends and the wider community ( Foucault, 1997). Reflective writing has the capacity for students to create positive social change ( Nelson, 2012); however it can be argued that rather than inspiring innovative thought and social change, it has now become an assessable exercise whereby student conform to what is expected in the rubric in order to pass the course. Without such strict criteria students may feel liberated and engage with broader social problems that are difficult to solve but interesting to reflect upon and explore.

## Verbalisation and the care of the self

Dental students are encouraged to reflect on their performance routinely during clinical experiences in verbal discussion with their educators. Such learning conversations allow students the opportunity to reflect on their experience with the aim of improving future performance ( Fejes, 2013). Foucault explored the integral role of verbalisation during confession, as one of the technologies of the self, describing it as “one of the main rituals we rely on for the production of truth” ( Foucault, 1978). As discussed earlier, uncovering the truth is an integral element in the care of the self. Evidence suggests that health professionals view reflective discussion as a necessary in developing better patient care ( Fejes, 2008), however the validity of reflection in determining competency is questionable ( Nelson, 2012). During clinical sessions, the reflective thoughts of students are verbalised to educators, placing them under constant under surveillance, which creates an overlap between technologies of power and self-care ( Fejes, 2013). Such conversations can be used to simply identify problems and pose solutions, which may result in achieving the desired grade, however to truly be reflective and care for one’s self these discussions should be confessional in that they reflect on their progress, the learning process, feelings and emotions ( Fejes, 2013). Such confessional practices may encourage a change in behaviour; Foucault described these exercises of self-care as “mastery over oneself..through the acquisition and assimilation of truth” ( Foucault, 1988).

## Critical reflection: self-care or surveillance tool?

One of the issues is whether the critical reflection is truly ‘self-care’ or whether it has moved to prompt students to look for what is right and what should be done. This is particularly evident when you consider the use of rubrics to assess a student’s self-reflection; educators need to consider whether they are truly using self-reflection in the curriculum for transformation and personal growth, or whether it is simply a surveillance tool ( Ng *et al.,* 2015). For students to be truly fit for practice they must see self-reflection as a way of being, rather than an assessment tool. Foucault indeed emphasized that to care for one’s self was a way of living ( Foucault, 1988). Students will not always have their educators to survey their clinical skills or knowledge, as such it is essential that they have the ability to critically reflect on their own professional competence to identify areas for development and growth.

Critical reflection may be encompassed in exercises other than reflective journal writing, including case presentations and clinical log books. The development of rubrics for the full range of assessments and examinations has compartmentalised knowledge, skills and behaviour in order to assess fitness to practice ( Park, Pelletier and Klingenberg, 2014). The accountability of fair and objective assessment seems to have gained precedence over fitness to practice, including care of the self and others. For example, when assessing medical history taking we often explore the student’s ability to collect relevant information and communicate this to their demonstrator, rather than assessing the interaction between student and patient. Much of the recent literature on clinical assessment focusses on how this can be authentic and objective, which has led to the development of Objective Structured Clinical Examinations (OSCEs), where actors are employed as simulated, standardised patients playing a designated role in a clinical scenario. In OSCEs the focus for the student is often the examiner, rather than the patient ( Johnston, 2014); these circumstances illustrate the panoptic schema that is described by Foucault as a mechanism of disciplinary power. The student’s awareness of being observed “compels them to structure their own behaviour in accordance with the power mechanism” ( Hoffman, 2011). Further, in such a panoptic mechanism, the educator’s responsibility to produce a competent graduate and their own professional reputation are at stake ( Foucault, 1995). This architecture results in a focus away from the care of the self and reflective practice, and towards the expected norms of the clinical skills and scientific knowledge within the discipline.

As higher education strives to produce competent graduates that are fit for practice, students learn what behaviours are acceptable and not acceptable within the profession. Educators “monitor progress, pass judgements and mould attitudes and behaviours” of students ( Mackey, 2007); as disciplined individuals they then self-regulate and conform to the expectations of the profession. Foucault attributed the success of disciplinary power to techniques of “hierarchical observation, normalizing judgement and the examination” ( Hoffman, 2011). The concern is that reflective practice has been reduced to an assessable exercise, rather than a way of caring for the self and others ( Nelson, 2012). This fear can be illustrated by the absence of the “person” in competency based curricula ( Park, Pelletier and Klingenberg, 2014), which results in a focus on the examiner and a lack of genuine care or emotion.

## Pastoral care and the care of the self

There is an increasing expectation for educators to providing pastoral care to students, with higher education institutions recognising that student wellbeing is being put at risk due to psychological stress and anxiety induced by poor academic coping strategies, unrealistic workloads and time pressures ( Van Dijk, Lucasson and Speckens 2015). Dental students in particular are at an increased risk of physical and psychosocial stress, with fear of failure, information-input overload, examinations, and shortages in allocated clinical time identified as contributing factors ( Naidu *et al.,* 2002; Thornton *et al.,* 2004). It is questionable how prepared educators are for this pastoral role, and how this is divergent from their disciplinary power. Foucault examined pastoral power and its historical roots in salvation which in the modern era came to include health and wellbeing ( Foucault, 1982). This type of power originated in religious institutions, but then expanded into the wider social arena to include family, medical practitioners and educators.

Student well-being requires pastoral care, given the mandatory reporting framework of the dental profession. The Australian Dental Council has outlined the required competencies of a newly graduated dental professional, and this clearly outlines the need to “recognise the importance of their own and others health and wellbeing on the ability to practise” (ADC, 2016). The regulations determined by the National Health Practitioner Regulation National Law describe that a practitioner may be guilty of misconduct if their actions are inconsistent with being a “fit and proper person to hold registration in the profession”; this includes mandatory reporting within and across the professions (including students) for notifiable conduct including intoxication by drugs or alcohol, sexual misconduct, and impairment. Such actions require reflecting on one’s own health and competence, which is consistent with the Foucault’s exploration of the care of the self. Foucault identified that “the care of the self isn’t another kind of pedagogy; it has to become permanent medical care” (1988). Here, reflective and confessional practice can be applied to both pastoral care and the care for the self to ensure fitness to practice.

## Conclusions

This paper has explored how the care of the self impacts on fitness to practice in dental education. Foucauldian concepts of disciplinary power, surveillance, governmentality and pastoral power have been applied to the dominant discourse of reflective practice, and bring into question whether this activity continues to promote care of the self or whether it has been reduced to an exercise used simply to assess the expected norms of the discipline.

## Take Home Messages

Educators need to carefully consider whether reflective practice has been reduced to an assessable exercise, rather than providing an avenue for students to exercise care for the self and others.

## Notes On Contributors

Dr Melanie Hayes is a Senior Lecturer in Interdisicplinary Education at The University of Sydney. ORCID iD: https://orcid.org/0000-0003-1001-1267
