# Supplementary material for: How does the ‘care of the self’ impact on fitness to practice in dental education?
Source: MedEdPublish (2016). 2019 Jun 20;8:139. [Version 1] doi: 10.15694/mep.2019.000139.1 (PMC10712450; doi:10.15694/mep.2019.000139.1)
Supplement: Peer review history

# Reviewer report: Reviewer response for version 1

**DOI:** 10.21956/mep.19868.r30022

**Published:** 2019-06-21

**Authors:** Michelle McLean

**Affiliations:** 1 Bond University

This review has been migrated. The reviewer awarded 5 stars out of 5 Thanks for reminding us about the need to challenge the 'why'. So often, we do and take things for granted because this is how it has always been done. I enjoyed reading this on a Friday afternoon after six weeks of intense 'teaching'. I would recommend this read for all educators in the health professions.

Reviewer Expertise:

NA

No decision status is available

# Reviewer report: Reviewer response for version 1

**DOI:** 10.21956/mep.19868.r30023

**Published:** 2019-06-21

**Authors:** Tan Nguyen

**Affiliations:** 1 Deakin University

This review has been migrated. The reviewer awarded 4 stars out of 5 This paper provides great discussion for the subject field. However, the argument made the author that questions whether 'reflective practice' may not promote 'care of the self' is weak given there are limited references specific to the dental profession, to support this statement. The care of self is one of many aspects in maintaining fitness to practice, which develops as a continuum from being a student in a health profession to lifelong learning when entering the health profession.

Reviewer Expertise:

NA

No decision status is available
